# Early life skin microbial trajectory as a function of vertical and environmental transmission in Bornean foam-nesting frogs

**DOI:** 10.1186/s42523-021-00147-8

**Published:** 2021-12-20

**Authors:** Sarah McGrath-Blaser, Morgan Steffen, T. Ulmar Grafe, María Torres-Sánchez, David S. McLeod, Carly R. Muletz-Wolz

**Affiliations:** 1 Department of Biology, University of Florida, 421 Carr Hall, Gainesville, FL 32611 USA; 2 Department of Biology, James Madison University, 951 Carrier Dr, Harrisonburg, VA 22807 USA; 3 Universiti Brunei Darussalam, Tungku Link, Gadong, BE 1410 Brunei; 4 North Carolina Museum of Natural Sciences, 11 West Jones Street, Raleigh, NC 27601 USA; 5 Smithsonian National Zoo and Conservation Biology Institute, Center for Conservation Genomics, 3001 Connecticut Ave., Washington, DC 20008 USA

**Keywords:** Amphibian, Amplicon sequencing, Bacteria, Brunei, Environmental transmission, Metabarcoding, Microbiome, *Polypedates*, Vertical transmission

## Abstract

**Background:**

The amphibian skin microbiome is an important mediator of host health and serves as a potential source of undiscovered scientifically significant compounds. However, the underlying modalities of how amphibian hosts obtain their initial skin-associated microbiome remains unclear. Here, we explore microbial transmission patterns in foam-nest breeding tree frogs from Southeast Asia (Genus: *Polypedates*) whose specialized breeding strategy allows for better delineation between vertically and environmentally derived microbes. To facilitate this, we analyzed samples associated with adult frog pairs taken after mating—including adults of each sex, their foam nests, environments, and tadpoles before and after environmental interaction—for the bacterial communities using DNA metabarcoding data (16S rRNA). Samples were collected from frogs in-situ in Brunei, Borneo, a previously unsampled region for amphibian-related microbial diversity.

**Results:**

Adult frogs differed in skin bacterial communities among species, but tadpoles did not differ among species. Foam nests had varying bacterial community composition, most notably in the nests’ moist interior. Nest interior bacterial communities were discrete for each nest and overall displayed a narrower diversity compared to the nest exteriors. Tadpoles sampled directly from the foam nest displayed a bacterial composition less like the nest interior and more similar to that of the adults and nest exterior. After one week of pond water interaction the tadpole skin microbiome shifted towards the tadpole skin and pond water microbial communities being more tightly coupled than between tadpoles and the internal nest environment, but not to the extent that the skin microbiome mirrored the pond bacterial community.

**Conclusions:**

Both vertical influence and environmental interaction play a role in shaping the tadpole cutaneous microbiome. Interestingly, the interior of the foam nest had a distinct bacterial community from the tadpoles suggesting a limited environmental effect on tadpole cutaneous bacterial selection at initial stages of life. The shift in the tadpole microbiome after environmental interaction indicates an interplay between underlying host and ecological mechanisms that drive community formation. This survey serves as a baseline for further research into the ecology of microbial transmission in aquatic animals.

**Supplementary Information:**

The online version contains supplementary material available at 10.1186/s42523-021-00147-8.

## Background

The amphibian skin-associated bacterial community (i.e., microbiome) plays a major role in organismal health and adaptation to biotic and abiotic factors [1–7]. Amphibians possess a unique interconnectedness with their microbial and macroscopic environment due to two important traits: the metamorphic process of many species and their semi-permeable skin. The quintessential amphibian life cycle and the transition of aquatic larvae onto land means that amphibian survival across life stages requires suitable habitat at each stage and increases the amount of habitat types required to support one organism. In addition, at each amphibian life stage gas exchange is facilitated via semi-permeable skin (obligate, facultative, or changing with life stage) opening up the host to greater environmental influence [8]. To avoid dehydration in terrestrial habitats and to facilitate cutaneous respiration most amphibian species require a persistent layer of mucus covering the skin which creates a hospitable environment for microbial proliferation [9]. Microbiota that inhabit this mucus layer are recognized as affecting host health [5]. Therefore, amphibians provide a unique circumstance in which to analyze the host-microbiome-environmental intersection.

Amphibians are no exception to the complex connectedness that all multicellular organisms have with their microbial community. These relationships can be mutualistic, commensal, or pathogenic in nature [10–12] and can shift depending on interaction dynamics and microbial transfer [8]. For instance, pathogenic microbes, such as the *Batrachochytrium sp.* chytrid fungi, can lead to high mortality in susceptible species while probiotics, or beneficial microbes, have been successfully utilized in amphibian disease mitigation efforts [2, 4, 13, 14]. Since the microbiome has been found to be a key factor in amphibian disease mitigation, research into the amphibian skin microbiome has emerged as a topic of considerable interest. The amphibian skin microbiome is influenced by a myriad of factors including genetics [15], life history [16, 17], behavior [18], physiology [19], environment [20–22], and exposure to introduced elements [23]. Combinations of these factors can influence the microbiome simultaneously, making it challenging to pinpoint sources of microbial disturbance and determine effective disease mitigation strategies [5]. Despite the complexity of microbial communities, commonalities have been found among the microbiomes of amphibian skin that can help elucidate how microbes become established on amphibian hosts. For instance, the cutaneous microbiota of amphibians have been found to be driven by selection processes, including host-species specific selection [15, 17, 24] and immune selection [25, 26]. One study found host ecology to be a driving factor in the amphibian cutaneous microbiome [16], suggesting that environmental microbial availability and diversity play a large role in establishing the amphibian skin microbiome. It is understood that changes in the microbiome accompany major developmental changes [17, 19, 27]. Few studies, however, have investigated the amphibian skin microbiome relative to changes in the organism during initial development—an important aspect of microbiome characterization. Microbial transmission can occur in a variety of ways including vertical transmission (parent to offspring), direct horizontal transmission (host to host), indirect horizontal transmission (host to environment to another host), and environmental transmission (environment to host) [28–30]. These modes of transmission, and/or a combination thereof, play a major role in host health and relative adaptability [31, 32]. Few studies, however, have directly compared modes of transmission and their influence on the microbiome [17, 29, 30].

Amphibian diversification includes a wide array of reproductive adaptations that have evolved to optimize offspring survival in a particular environment [33, 34]. Within that array are foam-nest breeding amphibians who have evolved a specialized behavior of depositing a foam nest containing fertilized eggs on structures (e.g., vegetation) overhanging a body of water [35–37]. To form the nest, females produce a secretion that both the females and males whip into a foam with their hind legs during amplexus. While the foam is being formed, females deposit eggs that the male simultaneously fertilizes, creating a frothy nest of fertilized eggs. Tadpoles hatch from eggs within the protection of the foam, and upon emergence from the nest, drop into the water below and continue the metamorphic process [38]. The foam is advantageous in that it helps protect the eggs and early tadpoles from predators [39–41]. This adaptive reproduction strategy evolved at least twice within the family Rhacophoridae and has been documented in 134 species of rhacophorid frogs [42]. Among these are tree frogs of the genus *Polypedates*, commonly found throughout Southeast Asia. *Polypedates leucomystax*, a species that commonly occurs in human-dominated landscapes, is the most well-known species of this genus. Kabisch et. al. [43] found the chemical composition of the *P. leucomystax* foam nest to be composed of 93% protein and 7% sugars. This high protein structural content allows nests to remain intact up to 1 month offering increased protection to the offspring inside [44]. Within nests, the tadpoles get a head start on development and are protected from many external pressures. Due to the contained nature of the offspring, foam nests provide an ideal system to begin exploring the question of how organisms acquire their skin-associated microbiota as environmental influences can be ruled out before the hatching stage. Therefore, *P. leucomystax*, and two congeners (*P. macrotis, and P. otilophus*) were chosen as model systems based on their reproductive mode, year-round breeding regimen [45], and availability at the study site (Kuala Belalong Field Studies Centre, Brunei, Borneo) [46].

In this study, we investigated the modality of microbial acquisition in offspring among three species of foam-nest breeding frogs (*P. leucomystax, P. macrotics, and P. otilophus*). Taking advantage of their specialized foam-nest breeding traits, we explored the question: Is the tadpole skin microbiome influenced more by initial reproductive contribution (vertical transmission) or the environment (environmental transmission)? This breeding mode also allowed us to follow changes to the skin microbiome as tadpoles transition to different surroundings (e.g., foam-nest to pond). We placed amplecting frogs from the wild in terraria in our field laboratory for sampling and transitioned tadpoles into in-field pond enclosures post-hatching. We predicted that the foam nest, in addition to protection, plays a critical role on establishment of the tadpole skin microbiome and hypothesized that these first microbial colonists would remain on the tadpole skin after the tadpole leaves the nest. To determine this, 16S rRNA metabarcoding of bacterial communities were conducted for samples taken from adults, tadpoles from inside the nest, leaves the nests were adhered to, water from inside the breeding terrarium, tadpoles after living in a pond environment for one week, and pond water. Our results provide the first characterization of the skin microbiome of the studied species and the first record of amphibian microbiomes from Borneo. This study provides insight into modes of microbial transfer within this specialized system, which is important for understanding influences on cutaneous microbial transmission and establishment.

## Results

### Microbial communities overview

**Fig. 1:**
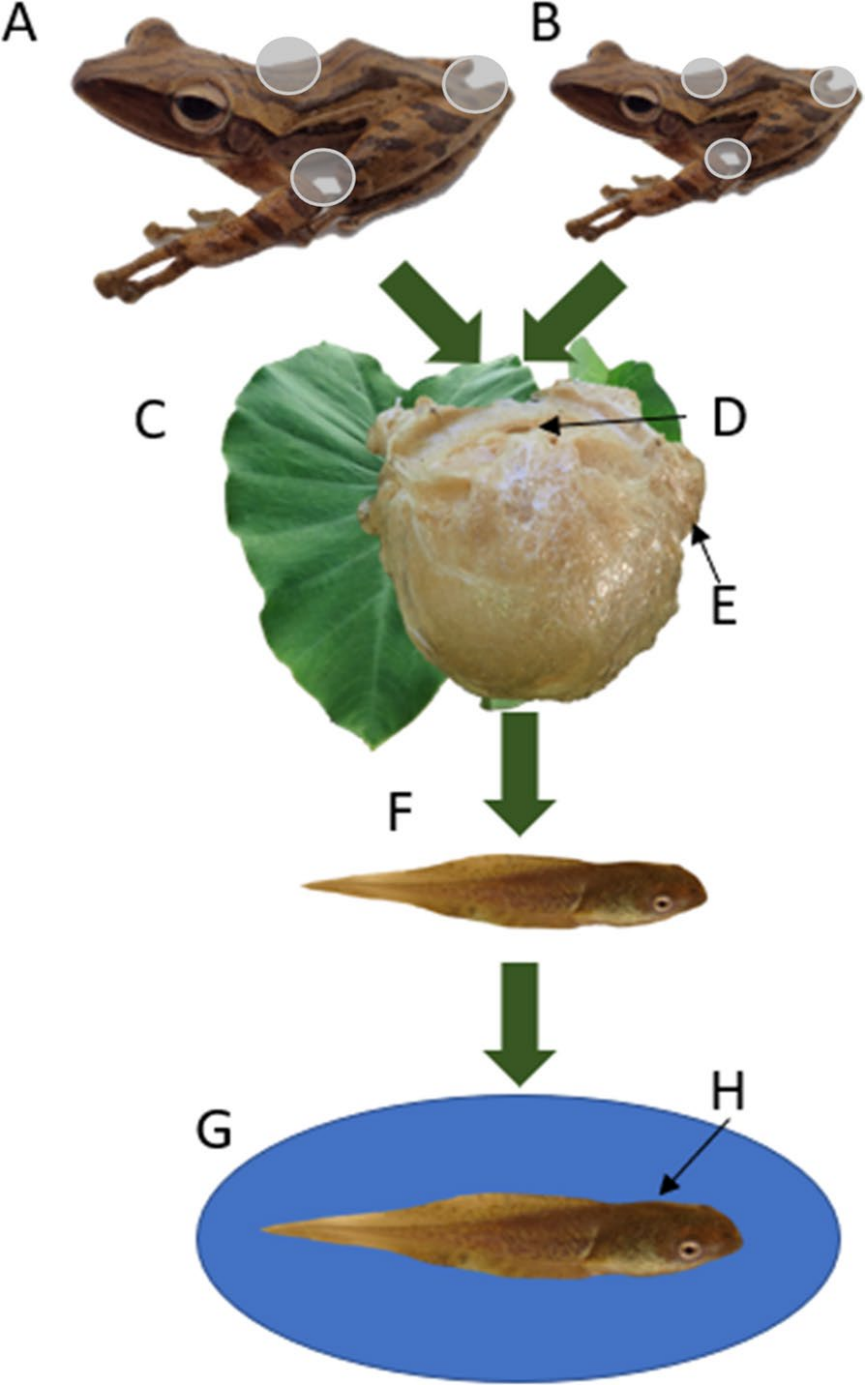
Visual representation of all variables sampled to characterize the microbial communities across a developmental and environmental gradient. **A** Adult female and **B** adult male individuals were sampled body location (grey dots; dorsal, ventral, cloaca). **C** Leaves were the attachment point for foam nests, which were sampled **D** inside and **E** outside for comparison. **F** Tadpoles extracted from the nest were compared to **G** water samples and **H** tadpoles after having one week of environmental interaction in a pond in-field enclosure. See Table 1 for the breakdown of the number of swabs per sample type

**Fig. 2:**
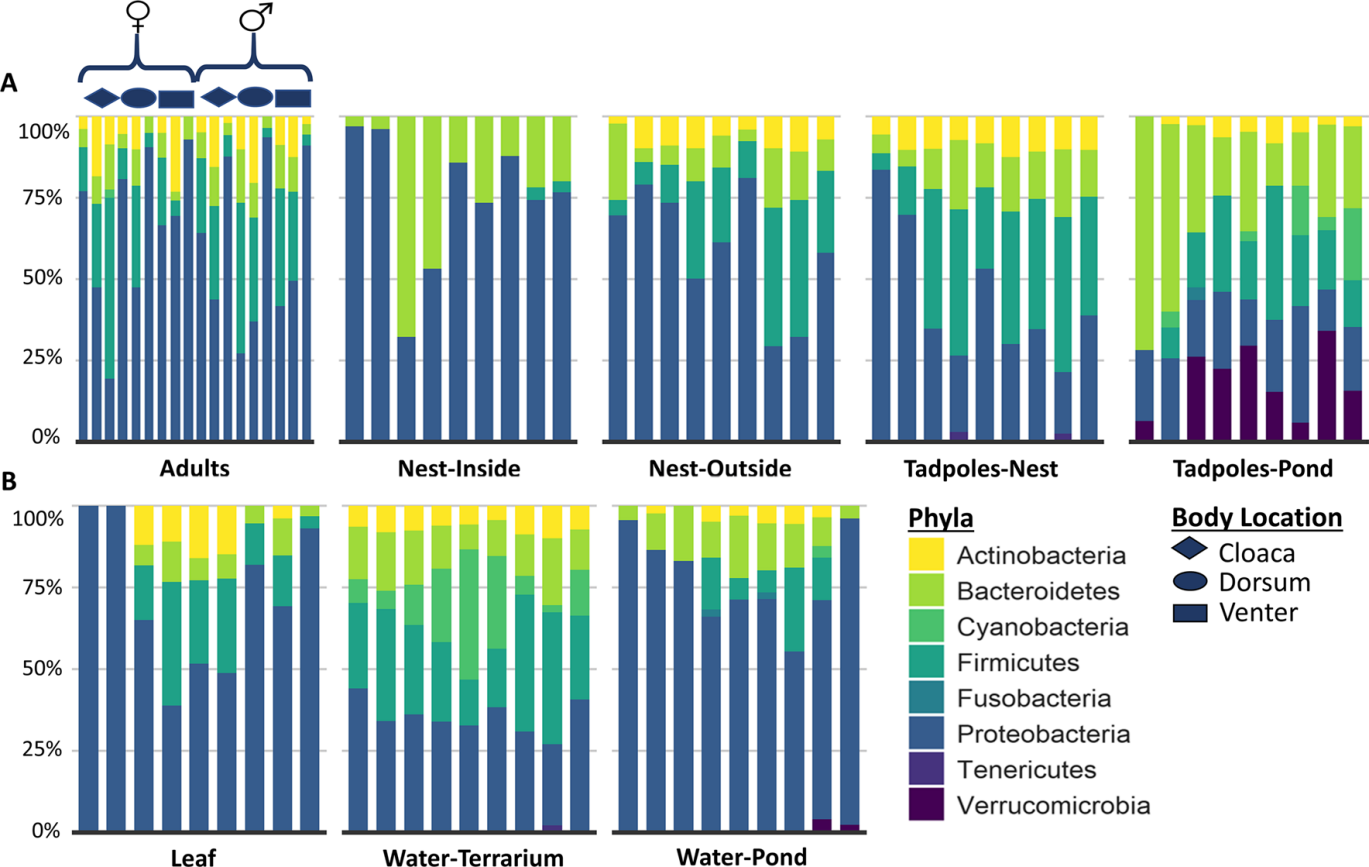
Taxonomic bar plots depicting the dominant phyla within the bacterial community of foam-nest breeding amphibians and their environment. Unassigned taxa and those with less than 2% relative abundance are not shown. Community composition for all replicates of each variable are presented at the taxonomic rank of phylum. **A** Amphibian specific samples: Organization of replicates for variable ‘Adult’ are arranged by sex and body location as denoted by the gender symbols and representative shapes. All other variables are in order by nest number (i.e., the first three bars are the replicates for nest 1, second three for nest 2, etc.). **B** Environmental samples: “Leaf” was the substrate the nest was adhered to. “Water-Terrarium” refers to the water at the bottom of the container in which adults made their nests and “Water-Pond” refers to water from the pond environment after tadpoles had one week of immersion

We had a total of 1,377,542 high-quality sequences that represented 2850 distinct operational taxonomic units (OTUs) from all environmental and amphibian-associated samples (n = 81; Fig. 1 and Table 1). Two archaeal phyla and 27 bacterial phyla were found to be present across all samples. Overall, Proteobacteria constituted 58% of all sequences followed by Firmicutes (15%), Bacteroidetes (13%), and Actinobacteria (5%). Environmental specific samples (leaf, n = 9; water-terrarium, n = 9; and water-pond, n = 9) and foam nest samples (inside, n = 9; outside, n = 9) had a higher percentage of Proteobacteria (62% and 67%, respectively) than amphibian adult and tadpole samples (45%). Contrastingly, amphibian associated samples (adults and tadpoles, nest) had more Bacteroidetes (16% and 18%, respectively) than the environment (10%). Firmicutes had higher percentages in amphibian adult and tadpole communities (18%) and the environment (15%) than in foam nests (10%) (Fig. 2).

**Table 1:** Representation of sampling design and depiction of the number of samples (n) collected for each category

Species	Nest		Body site for each adult (male/female pair per species)			Tadpoles from nest	Environmental samples of nest		Tadpoles from pond	Environmental samples of pond
	Interior	Exterior	Dorsal	Ventral	Cloaca		Leaf	Water (terrarium)		Water (pond)
*Polypedates leucomystax*	3*	3*	2	2	2	3	3*	3	3	3
*P. macrotis*	3*	3*	2	2	2	3	3*	3	3	3
*P. otilophus*	3*	3*	2	2	2	3	3*	3	3	3
Total	9	9	6	6	6	9	9	9	9	9

We collected three samples within each nest from different areas of the nest interior and exterior as well as different areas on the leaf to which each nest was attached (denoted by an asterisk) and considered them independent samples as they represented distinct nest or leaf microhabitats. Each nest sampled consists of a pair of adults, nest, environment, and tadpoles both before and after environmental interaction (Graphical representation of sampling design, Fig. 1)

### Characterization of the Polypedates microbiome and their environment

Amphibian specific skin-associated bacterial communities consisted of 512,532 high-quality sequences that represented 2521 distinct OTUs from only the amphibian samples (adults, n = 18; tadpoles [before and after environmental interaction], n = 18; Table 1). Proteobacteria dominated the skin bacterial phyla constituting approximately 45% of sequences followed by Firmicutes (18%), Bacteroidetes (16%), Actinobacteria (7%), Verrucomicrobia (5%), and Cyanobacteria (2%) (Fig. 1A). To determine if our amphibian microbial communities differ from those of other amphibians, we compared our dataset to a global dataset of amphibian microbiomes [21]. We found that the three Bruneian frog species we sampled were host to 31 unique genera not detected on any of the 205 amphibian species sampled in 13 countries previously (Additional file 1: Table S2). Of these distinct genera only one, *Bordetella*, is present among the top five most abundant representing 3% of amphibian-associated bacterial sequences. The top five most abundant genera on these frogs were *Acinetobacter* (10%), *Flavobacterium* (4%), *Pseudomonas* (4%), *Bordetella*, and *Lactobacillus* (2%), with the majority of the community being composed of low abundance taxa, including most genera unique to our Bruneian amphibian samples (Additional file 1: Fig. S1).

Adults differed in microbiome composition among host species, but tadpoles did not. Adults had dissimilar microbiomes among species (PERMANOVA–Jaccard pseudo-*F*_(2,15)_ = 3.046, R^2^ = 0.289, *p* = 0.001; Bray–Curtis pseudo-*F*_(2,15)_ = 8.353, R^2^ = 0.527, *p* = 0.001; Pairwise PERMANOVAs *p* < 0.05 for all comparisons). However, there was no indication of species level effects at the tadpole stage, either when sampled from the nest or from the environment (Tadpole-nest: PERMANOVA (df = 2) – Jaccard pseudo-*F*_(2,6)_ = 1.357, *p* = 1; Bray–Curtis pseudo-*F*_(2,6)_ = 2.537, *p* = 1; Tadpole-pond: PERMANOVA–Jaccard pseudo-*F*_(2,6)_ = 1.325, *p* = 1; Bray–Curtis pseudo-*F*_(2,6)_ = 2.591, *p* = 1). Within adults, there were no significant differences between sex or among body locations in microbiome composition (Pairwise PERMANOVAs Jaccard and Bray–Curtis for Sex and Body Location, *p* > 0.05 for all comparisons).

Foam nest microbial communities (nest-inside, n = 9; nest-outside, n = 9; Table 1) consisted of 244,169 total sequences representing 1744 OTUs. Compared to adult and tadpole amphibian samples, nest samples contained a higher percentage of similar top phyla including Proteobacteria (67%), Bacteroidetes (18%), Firmicutes (10%), Actinobacteria (4%), and Tenericutes (0.4%). There was no significant difference between frog species for either the inside or outside of nests (PERMANOVA, Nest-in: Jaccard pseudo-*F*_(2,6)_ = 7.754, *p* = 1, Bray–Curtis pseudo-*F*_(2,6)_ = 5.347, *p* = 1; Nest-out: Jaccard pseudo-*F*_(2,6)_ = 1.542, *p* = 1, Bray–Curtis pseudo-*F*_(2,6)_ = 3.331, *p* = 1). However, significant differences existed between nest sampling location (e.g., nest inside versus nest outside) (PERMANOVA: Jaccard pseudo-*F*_(1,16)_ = 4.977, R^2^ = 0.237, *p* = 0.001, Bray–Curtis: pseudo-*F*_(1,16)_ = 4.185, R^2^ = 0.207, *p* = 0.001).

**Fig. 3:**
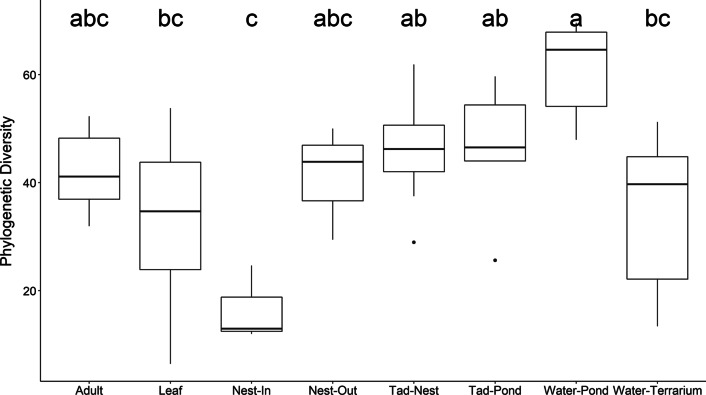
Boxplots comparing Faith’s phylogenetic diversity by sample type based on Tukey HSD post-hoc results from ANOVA significance values. Letters at the top of each boxplot denote significant differences. Nest interior samples have the lowest phylogenetic diversity overall with the highest variance displayed by water from the pond environment

Amphibian bacterial communities differed throughout the life cycle and differed from environmental samples in alpha diversity metrics (within-sample diversity). Environmental and amphibian-specific samples showed a wide variation in alpha diversity with significant effects by category (e.g., adult, tadpole-nest, nest-inside, tadpole-pond, etc.) (species richness (SR) ANOVA: F_7,50_ = 6.997, *p* < 0.001 and Faith’s phylogenetic diversity (PD) ANOVA: F_7,50_ = 8.709, *p* < 0.001). The lowest alpha diversity metrics were from the nest interior (SR: mean = 145, ± 126.58; PD: mean = 16.51, ± 7.04) whereas water from the pond environment had the highest (SR: 1011.78 ± 173.51; PD: mean = 60.72 ± 8.28). Tadpoles had similar alpha diversity before and after environmental interaction but were significantly different from adults. Adults had alpha diversity metrics closer to that of the nest exterior (Fig. 3). Nest exteriors were higher in species richness and microbial diversity than nest interiors (Nest inside – SR: mean = 145 ± 126.58; PD: mean = 16.51 ± 7.04; Nest outside–SR: mean = 655 ± 243.47; PD: mean = 41.08 ± 10.55) and tadpoles sampled after environmental interaction were significantly different from the pond environment (Tadpole pond–SR: mean = 700.67 ± 209.06; PD: mean = 47.03 ± 9.86; Water pond–SR: 1011.78 ± 173.51; PD: mean = 60.72 ± 8.28).

**Fig. 4:**
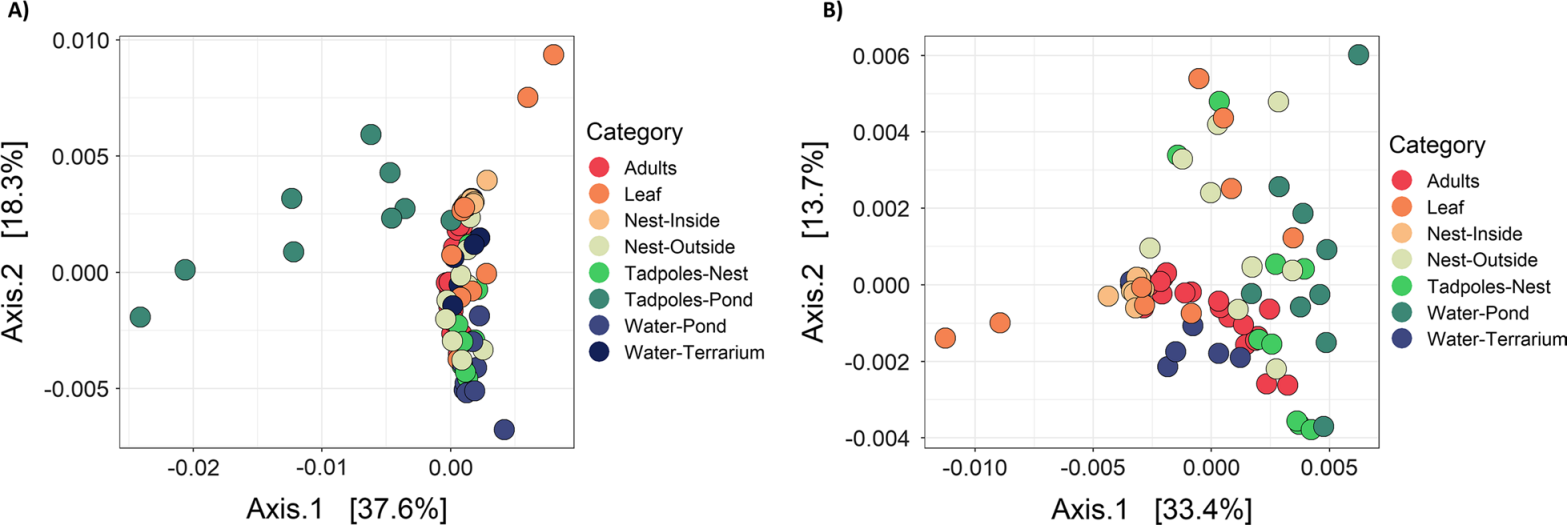
Principal Coordinate Analysis (PCoA) plots comparing the beta diversity of all sample replicates by weighted UniFrac distance matrices. **A** All sample categories including tadpoles after one week of interaction with pond water and **B** sample categories excluding tadpoles after pond water interaction. This represents the compositional differences between samples in low-dimensional space based on bacterial phylogenetic relatedness and abundance

Amphibian and environmental samples also differed in beta diversity metrics (between-sample diversity, Fig. 4). Statistically significant differences were found among sample categories (e.g., adult, tadpole-nest, water-pond, etc.) (PERMANOVA: Jaccard pseudo-*F* = 2.127, R^2^ = 0.229, *p* = 0.001, DF = 7; Bray–Curtis pseudo-*F* = 3.459, R^2^ = 0.326, *p* = 0.001, DF = 7; unweighted UniFrac pseudo-*F* = 2.265, R^2^ = 0.241, *p* = 0.001, DF = 7; weighted UniFrac pseudo-*F* = 5.583, R^2^ = 0.439, *p* = 0.001, DF = 7) so pairwise comparisons were performed to determine differences for specific categorical comparisons (Additional file 1: Table S1). Adult microbial communities were similar to microbial communities on nest exterior, on terrarium leaves (all metrics: *p* > 0.05), and generally in the nest interior (all metrics, but unweighted UniFrac: *p* > 0.05), on tadpoles in the nest (all metrics, but Bray–Curtis: *p* > 0.05), and in the terrarium water (all metrics, but weighted UniFrac: *p* > 0.05). Adult microbial communities were distinct from microbial communities on tadpoles in the pond and in the pond water (all metrics: *p* < 0.05). Tadpole microbial communities in the nest were similar to microbial communities on the adults and nest exterior (all metrics: *p* > 0.05), but distinct from the nest interior, terrarium leaves, and the terrarium water (all metrics: *p* < 0.05, Additional file 1: Table S1). Tadpole microbial communities in the pond were distinct from all other microbial communities, including adults, tadpoles in the nest and pond water (all metrics: *p* < 0.05). Overall, we found that tadpoles from the nest were similar to adults and foam nest exteriors, and that tadpoles after pond water interaction had their own distinct skin microbiomes (Fig. 4).

### Analysis of vertical and environmental transmission

**Fig. 5:**
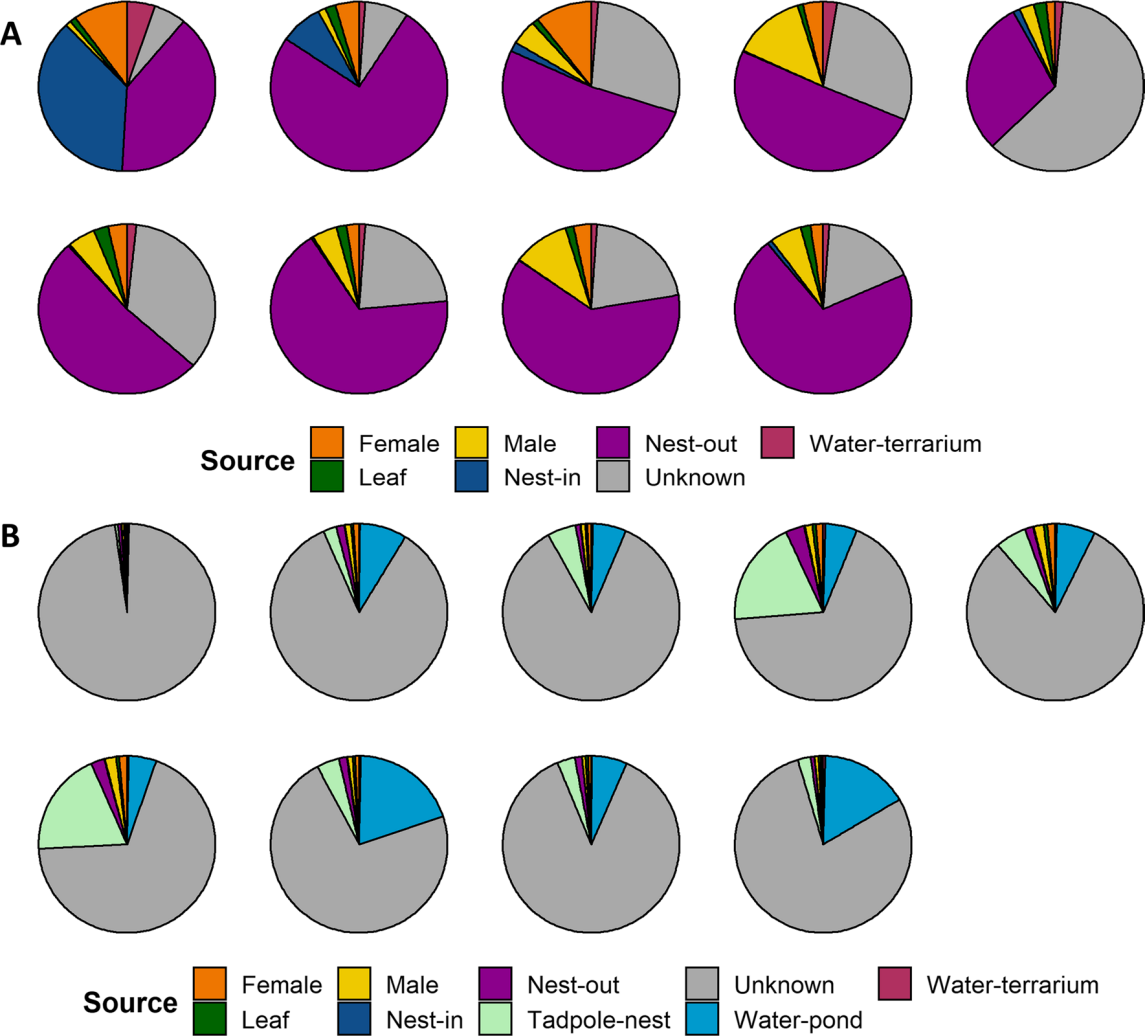
SourceTracker analysis results showing the percentage of microbial influence contributing to each tadpole’s skin microbiome. **A** Microbial sources contributing to tadpoles in the nest, **B** microbial sources contributing to tadpoles after one week of pond water interaction

We found evidence for both vertical and environmental transmission, with vertical being more impactful on the microbiome. We used multiple statistical and analytical approaches to support this finding. First, to examine whether the tadpole skin microbiome was influenced more by parentally or environmentally derived microbes we focused on differences in tadpoles sampled before leaving the nest and after one week of pond water interaction. We found using SourceTracker analysis that a large percentage of microbial sources were known (average 80%) for tadpoles in the nest, while a small percentage of microbial sources were known (average 25%) for tadpole microbial communities after pond water interaction (Fig. 5). Of the identified sources, the tadpole skin microbiome in the nest was attributed mostly to the nest exterior and adults with only one tadpole demonstrating a relatively high proportion of OTUs attributed to the nest interior (Fig. 5A). Tadpoles after pond water interaction had a high percentage of unknown OTU sources with small percentages attributed to the pond water and tadpoles from the nest (Fig. 5B).

**Fig. 6:**
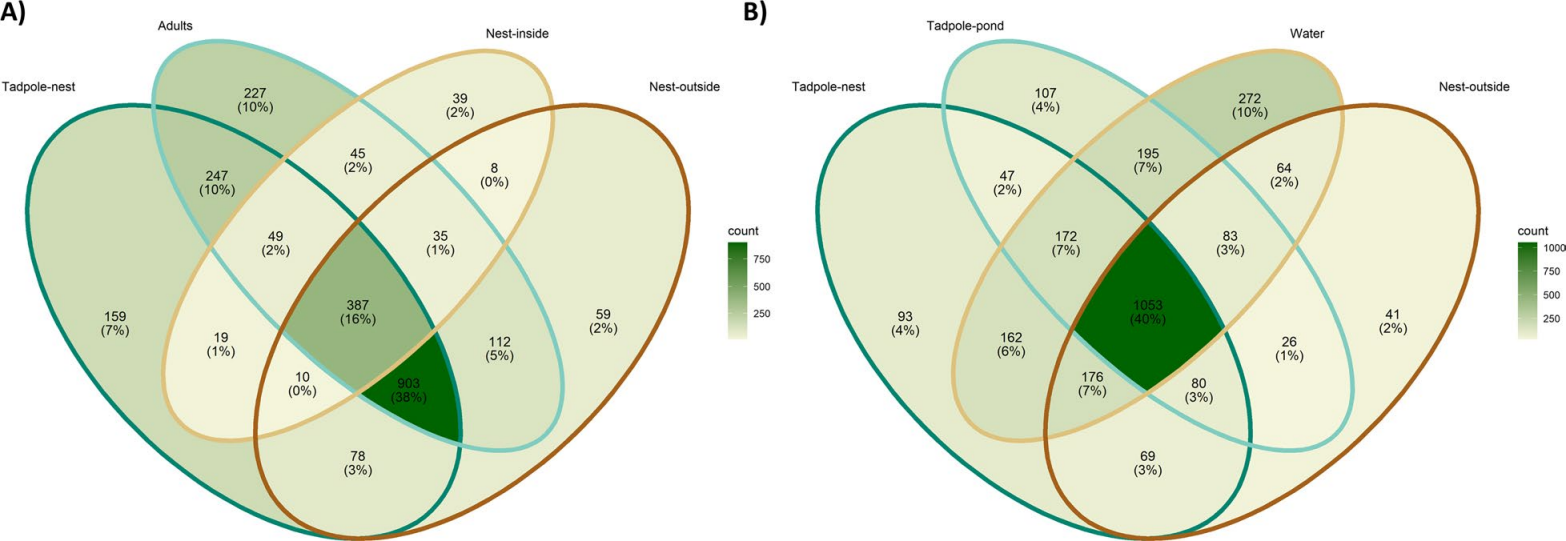
Venn diagrams of shared numbers and proportions of OTUs among categories. **A** Samples associated with vertical transmission and **B** samples associated with environmental transmission

Next, we compared shared OTUs among sample categories. To bin samples as contributing to vertical transmission, we compared adults, nest interiors, nest exteriors, and tadpoles sampled from the nest. To identify evidence for environmental transmission, we compared tadpoles sampled after environmental pond water interaction as well as compared those to tadpoles before pond water interaction and to nest exteriors. We found that tadpoles sampled before environmental interaction shared the most OTUs with adults and the nest exterior (Fig. 6A: 38% shared OTUs). Contrary to our prediction, these tadpoles shared the least amount of OTUs with the nest interior (Fig. 6A: 1% shared OTUs). Tadpoles after one week in the pond environment shared a high number of OTUs with pond water, nest exteriors, and tadpoles from the nest (Fig. 6B: 40% shared OTUs). This was more than the numbers shared solely between tadpoles from the pond and tadpoles from the nest (Fig. 6B: 2% shared OTUs) and pond tadpoles and the nest exterior (Fig. 6B: 1% shared OTUs). In comparing vertical and environmental categories of shared OTUs, vertically influenced microbial communities had a roughly 4.5-fold higher amount of shared OTUs compared to environmentally influenced communities (903/195 OTUs respectively). The number of shared taxa show evidence for both vertical and environmental influence, with more support for vertical transmission continuing to influence the tadpole microbiome after one week of environmental immersion.

To determine whether there were any specific OTUs that remained at relatively abundant levels across categories of vertical and environmental transmission we used differential abundance analyses to identify OTUs of similar abundances on adults, tadpoles in the nest, and tadpoles after pond water interaction. Pairwise comparisons between the tadpole skin microbiome, both in the nest and in the pond, and the adult, nest interior, nest exterior, and pond water microbiomes allowed us to find OTUs with abundances one or more units of fold change different between categories (Additional file 1: Table S3 and Figure S2). The biggest difference was found between the skin microbiome of the tadpoles inside the foam nest and the microbial community of the foam nest inside. From the OTUs found at a higher abundance on the skin of the tadpole inside the nest, 448 OTUs were found in similar abundances in the adult skin microbiome and remained in high abundance on the skin of the tadpoles after pond water interaction. These OTUs, abundant in amphibian samples, are candidates for vertical transmission and potentially important contributors to amphibian skin function due to their persistence in the tadpoles across environmental shifts. Among these candidate OTUs, we identified four Archaea (two members of Methobacteriaceae and two of Methanomassiliicoccaceae) and twelve different phyla of bacteria including Firmicutes (46% of candidate OTUs), Proteobacteria (17%), Bacteroidetes (15%), and Actinobacteria (15%).

### Technical replicates

We found that while some technical replicates were dissimilar to one another, this had minimal impact on our biological conclusions. We took three technical replicate swabs for each independent samples (Table 1, see more details in “Methods”) and applied a triangulation test [47] to determine if technical replicates were drawn from the same distribution. In the triangulation test, we failed to reject the null hypothesis that replicates were drawn independently, indicating that replicates within samples were not always more similar to one another than to replicates from other biological samples. However, we wanted to verify if this dissimilarity affected our biological conclusions. We chose a random sample from each technical replicate set, analyzed community composition differences among environment and amphibian-specific sample types with Pairwise PERMANOVAs for Jaccard (presence/absence of taxa), Bray–Curtis (presence and abundance of taxa), unweighted Unifrac (phylogenetic relationships), and weighted Unifrac (phylogenetic relationships and abundance) distances, and repeated this random subset for each subsequent technical replicate until all replicates had been compared. Overall, 4 out of 28 comparisons showed significant differences for only one replicate compared to the other technical replicates for a given distance matrix (Additional file 1: Table S1), indicating that the majority of technical replicates produced similar biological results. Significant differences reported herein between sample types were only considered significant if at least two replicate comparisons had a *p* value < 0.05.

## Discussion

Host-associated microbial characterizations are increasing with the ease and feasibility of data collection and sequencing processes [46]. However, some taxa and geographic regions have been favored in this endeavor, leaving other areas unexplored [48]. This led us to examine the first skin-associated microbiome characterization of amphibians from the island of Borneo. The focal organisms for this study were also selected due to their specialized breeding strategy of producing a foam nest that encapsulates eggs and early-stage tadpoles. With this confined system we were able to investigate modes of microbial transmission and their impact on the early life stages of these organisms. It is understood that microbes play an important role in host health, but initial establishment of the host-associated microbiome has been more difficult to discern [5]. This amphibian model provided insight into the relative influence of vertical versus environmental microbial transmission on early-stage tadpoles.

Our results support a greater relative influence of vertical transmission on the tadpole skin-microbiome with a secondary shift once they interact with the external environment. Although sample size was limited due to finite sampling materials and field constraints, our results reflect patterns of transmission. Once the nest was built, adult frogs shared similar microbial communities to the foam nest outside and the tadpoles inside the nest. Contrary to our prediction that the foam nest provides microbial seeding as well as protection, host specific factors enable tadpoles inside the nest to harbor a distinct microbial community that does not mirror its interior foam nest environment but more closely resembles adults and the hardened nest exterior. The tadpole skin microbiome being similar to the adult microbiome is in congruence with other amphibian investigations into vertical transmission that take into account behaviorally mediated parental care [30, 49, 50]; however, in our unique system parental care is replaced with a “nest-care” proxy. While direct parent-to-egg contact behavior seems to facilitate microbial transfer, our findings underscore those found by others that non-behaviorally facilitated transfer and host-specific factors are drivers of the tadpole skin microbiome over environmental influence [27, 31]. Tadpoles only have innate immunity at this early stage, but even basic immunological responses can affect the microbiome [26, 51]. Therefore, it seems that underlying mechanisms (e.g., potential immune response selection) are what determine the early-life tadpole skin microbiome, not the tadpole’s immediate surroundings of the foam nest. Bacterial abundance on tadpole skin was different from the abundances of the same OTUs on adult skin except for the cloacal microbiome. This provides evidence for the relative importance of the cloaca in offspring microbial acquisition, similar to what has been documented in other vertebrates [52–55]. No differences were determined between male or female cloaca, which might reflect the reproductive mode of external fertilization. Other research into foam-nest breeding amphibians has demonstrated that females are the source of the secretion that allows for foam nest formation [44]. We suggest future research focus on the role of transmission from the cloaca, from females to offspring, and the potential for internal transfer during egg formation and brooding [27].

Tadpoles sampled from the nest shared a large number of OTUs with the foam nest exterior and nest microbial communities differed greatly between inside and outside of nest. Differences in nest microbial community by location could possibly be due to the physical and chemical properties associated with nest interior and exterior sites. The interior stays foamy and moist while the exterior hardens, possibly contributing structure for microbes to grow or shielding the nest interior from external environmental microbes to which it is more exposed. Nest interior chemical composition is made up of mostly surfactant proteins [43, 56]. Similar proteins described from a related Japanese rhacophorid species (*Rhacophorus arboreus*) had antimicrobial properties [57]. This could possibly account for the relatively low alpha diversity of the foam nest interiors (Fig. 3) and is further supported by observations that these nests maintain a moist homeostasis and do not become overrun with environmental fungi or biofilms, but further research is needed to confirm. Also, proteins homologous with keratin were characterized from the *Rhacophorus arboreus* foam nest [57], which may or may not contribute to the structure of the foam nest exterior seen in our species. If so, this would provide evidence for the similarity between nest exteriors and adults. The tadpoles however do not yet have keratinized skin, and host specific factors need to be examined to determine drivers of selection for the initial tadpole skin-associated microbial community.

Tadpoles after pond water interaction demonstrated a shift in their skin-associated microbial community that did not reflect that of the pond water environment, nor did it show similarities to their nest-sampled counterparts. SourceTracker analysis showed a higher percentage of an unknown microbial source for these tadpoles compared to those sampled from the nest (Fig. 5). This result reflects underlying ecological mechanisms, such as microbial species interactions (e.g., competition) and increased abiotic influences of the pond water environment (e.g., temperature, light) compared to the nest interior, that might shift the bacterial OTUs present as well as their abundances. Other amphibian systems show a demonstrated shift in the skin-associated microbial community across development [5, 17, 19, 27], even across hatching [27], but there is widespread evidence that amphibian and more generally vertebrate skin microbiomes are not solely determined by their environmental pool of microbes [27, 48, 49]. This is also underscored by our findings that some higher abundance bacterial taxa persist across environmentally induced shifts and remain abundant in the host skin microbiome. It is likely that both deterministic and stochastic processes are occurring that drive skin microbiome assembly [48]. For instance, environmental interaction is where adaptive immunity can begin to form [51], which might influence the host-associated microbial community and subsequently host health [58–60]. Although elucidating these interactions is outside the scope of this study, there is evidence for various potential drivers of host-microbial-environmental selection in this context [6, 15, 16, 25, 30, 61, 62]. Determining drivers of these types of ecological selection is complex and therefore we suggest future related research with these study species focus on ex situ laboratory experiments. Naturalized variables can be more controlled, as demonstrated in this study that allowed for as naturalized a transmission event as possible and controlled for external elements, such as accidental sampling of conspecifics.

Our study is the first to report amphibian associated microbial communities from the island of Borneo or the country of Brunei, and we found unique patterns not seen in other global amphibian microbiome datasets [21]. Notably, the genus *Bordetella* was one of the most abundant genera among our amphibian bacterial communities. *Bordetella petrii* is an environmental as well as a host-restricted and pathogenic *Bordetella* strain which has not previously been documented in association with amphibians [63]. Ultimately, 31 new amphibian candidate genera were found to exist on our *Polypedates* frogs from Brunei. This highlights the general lack of understanding of microbial communities from remote regions and especially those associated with unique hosts (e.g., foam nesting frogs).

We show that vertical transmission, environmental transmission and host regulation in early life all play a role in shaping amphibian skin microbiomes. Amphibians serve as important ecological components across a wide range of ecosystems [64]. Unfortunately, many are currently experiencing habitat loss, climate change, and infectious disease [65–67]. For instance, fungal pathogens that cause the disease chytridiomycosis have driven some of the largest declines of any vertebrate taxa in history [65]. Declines have escalated to include 43% of all described amphibians, a number that is likely conservative [10, 68]. These losses cause ecological cascades [64] resulting in a plethora of yet uncharacterized effects. With this loss of biodiversity, we are not only losing many charismatic and interesting species, but also adaptations, physiological specializations, uncharacterized species, and specialized microbial hosts that are central to further understanding the natural world. Without a better understanding of the microbial community ecology associated with these host types, we are undoubtedly losing untold numbers of microbial entities with each host extirpation as well.

## Conclusion

Amphibian skin-associated microbiomes are diverse communities that play an important role in host health [5, 60, 69]. Microbial transmission is important to delineate in order to understand how amphibian offspring acquire their initial microbiome, which can have profound health effects for them later in life [13, 23, 60, 69]. Gaining an understanding of the processes attributed to microbial acquisition can aide in amphibian conservation efforts, particularly those focused on probiotic efforts to reduce the effects of chytridiomycosis. The conservation implications of this study also may be transferrable to other tropical areas, including the New World tropics and Africa, where species convergent for this breeding strategy exist [42, 70, 71]. This type of exploration is novel for old-world tropical amphibian species and should be expanded within this region.

## Methods

### Study site

All samples were collected in the field May–July 2017 from two sites at the Kuala Belalong Field Studies Centre (KBFSC) in Ulu Temburong National Park, Brunei (Borneo). The first was a permanent man-made pond located in disturbed secondary forest across the Temburong river from Ulu Ulu Resort, upriver from KBFSC (lat: 4.555807 N, long: 115.153628 E). At the time of our work, the pond measured approximately 5 m wide, 9 m long, and 1.5 m at maximum depth with dark, tannin-rich water and a substrate of fine sediment covered by dense vegetative detritus. Woody shrubs, herbaceous vegetation, vines, and trees surrounded the pond. The forest canopy reached 4 m high over the pond and was mostly closed with a few open patches where trees had fallen. During the collection period, six species of amphibians were observed in or around the pond, including; *Polypedates macrotis, P. otilophus, Limnonectes cf. kuhlii, L. leporinus, Rhacophorus pardalis,* and *Occidozyga laevis*. The second site, a 1.5 m diameter concrete basin with 0.5 m maximum water depth and approximately 10 cm of fine silt covered by a layer of detritus at the bottom, was located at KBFSC and part of a water drainage system used to divert rain water away from buildings (lat: 4.546525 N, long: 115.158038 E). The canopy over the basin was open. Mixed vegetation reaching 1 m high and overhanging part of the water was present on one side of the basin, including pandan (*Pandanus amaryllifolius*). During the collection period *P. leucomystax* and *P. otiolophus* species were observed in or around the basin.

### Sample collection

This study was approved by the Institutional Animal Use and Care Committee of James Madison University (A15-15) and was completed with permission from the Universiti Brunei Darussalam (UBD/AVC-RI/1.21.1[a]). We collected one breeding pair for each of three species for sampling: *Polypedates leucomystax* (four-lined treefrog), *P. macrotis* (dark-eared treefrog), and *P. otilophus* (file-eared treefrog). These three species occur syntopically in Ulu Temburong National Park. The collection and sampling of more pairs from each species was limited by the number of supplies (i.e., sterile swabs) and the remoteness of the field location. Amplectant pairs of frogs were collected during nocturnal surveys conducted 18:00–23:00 h. Pairs in amplexus were captured by hand with clean nitrile gloves and placed into a clean plastic bag for transport to the lab at KBFSC. Once back at the lab pairs were placed in individual plastic terrarium (approximately 30 cm × 25 cm × 50 cm) previously sterilized with 100% ethanol. The first pair of frogs collected and placed in a bare terrarium did not re-engage in amplexus. To facilitate the mating process, approximately 2 cm of water from the concrete basin and one to two large leaves from the same location were added to all terraria. As the first pair was found to forego amplexus and complete nest formation without these naturalized conditions, the same process was repeated for subsequent pairs for consistency.

Once pairs had completed nest construction, each adult individual was removed from the terrarium using new sterile nitrile gloves. Individuals were rinsed with 100 ml of distilled water before swabbing to ensure sampling of amphibian skin-associated microbes rather than transient microbes or environmental material [24, 72, 73]. New bottles of Suci brand distilled drinking water (Suci Mas Company, Bandar Seri Begawan, Brunei Darussalam) were used for each individual specimen to decrease inter-sample contamination. Each specimen was swabbed for 15 s at each of three different body locations (cloaca, dorsum, and venter) using one sterile rayon swabs for each location (MW113, Medical Wire Equipment & Co. Ltd., Corsham, UK) [15]. To ensure that individuals were not re-sampled and to provide whole voucher specimens for additional studies, specimens were euthanized in a dilute solution of MS-222, fixed in 10% neutral-buffered formalin, and later transferred to 70% EtOH. Liver tissue samples taken prior to fixation were stored in RNAlater. All specimens were deposited at the North Carolina Museum of Natural Sciences (NCSM; Additional file 1: Table S4).

Approximately one week after collection (when movement was first observed in the nest), we sampled the rest of the contents in the terraria including nests, leaves, water, and tadpoles from inside the nest. Due to differences in consistency between moist nest interiors and crusty nest exteriors we sampled both nest surfaces. Exterior surfaces were sampled by rubbing a sterile swab over the surface of the nest for 15 s. The interior was sampled by gently creating an opening indentation with a gloved finger and inserting a sterile swab, moving it around within the nest for 15 s. All foam nest samples were completed in triplicate. Second and third interior foam nest sampling swabs were inserted via the same opening to maintain nest integrity, but sampled in different directions. To sample tadpoles while still inside the nest, a sterilized 15 mL plastic pipette with the tip cut (to allow for extra space) was inserted into the indentation and several tadpoles were extracted. Tadpoles were then deposited into a single sterile, plastic container and rinsed in the same manner as the adults with Suci brand distilled water. We randomly chose three tadpoles from the container and swabbed each individual ventrally and dorsally for 15 s, equating to roughly 5–7 strokes on each side [29]. Once all of the remaining tadpoles from the nests hatched (approximately 10 days later), they were placed in in-field enclosures consisting of 45.72 cm × 66.04 cm mesh laundry hampers with mesh, zippered lids (Collapsible Laundry Hamper, Whitmor Inc., Southaven, MS, USA) inside the concrete basin at KBFSC. Each enclosure contained larvae from a single clutch and allowed for maximum interaction with the environment while containing the tadpoles and protecting from predation and contact with other tadpoles living in the basin. One week after placement, pond water was sampled by inserting a swab approximately 10 cm into the water column within the enclosure and moving at this depth for 15 s [17]. Then, tadpoles were removed from enclosures via a sterilized dipnet, placed in a sterile plastic container, and taken back to the KBFSC lab where the same swabbing protocol was followed as for the tadpoles extracted from the nest. All sampling was completed in triplicate. See Table 1 for information regarding sample sizes for all variables.

After sampling, all swabs were immediately placed in sterile 1.5 ml Nalgene cryotubes (Thermo Fisher Scientific, USA), stored inside vacuum insulated canisters (Rambler 64 oz & 36 oz, Yeti Coolers LLC, Austin Texas, USA) and placed in a − 20 °C freezer. Samples remained frozen in the vacuum insulated canisters (packed with additional ice-filled cryotubes) during approx. 30 h of travel and were subsequently transferred to a − 80 °C freezer at James Madison University until processing.

### Sample processing

Amplicon sequencing of the 16 s rRNA gene was used to determine bacterial community structure for all amphibian and environmental variables. DNA was extracted using the DNeasy PowerSoil Kit (Qiagen, Valencia, CA, USA) according to the manufacturer’s protocol. The V4 region of the 16S rRNA gene was PCR-amplified with barcoded primers following the 16S Illumina Amplicon Protocol standard for the Earth Microbiome Project (515f/806r) [74]. Each 25 μL PCR contained: 6.5 μL molecular grade PCR water, 12.5 μL 5 Prime Hot Master Mix, 0.5 μL each of the forward and reverse primers, and 5 μL genomic DNA. PCR conditions were: denaturation for 3 min at 94 °C, amplification for 35 cycles of 45 s at 94 °C, 60 s at 50 °C, and 90 s at 72 °C, and a final extension of 10 min at 72 °C. Amplified samples were run on a 1% agarose gel to check for amplicons and then cleaned using AMPure XP Beads (Beckman Coulter, Inc., Brea, CA, USA). Unique dual indices were then added to each cleaned product in a second PCR step. For this round, each 50 μL PCR contained: 25 μL 5 Prime Hot Mastermix, 5 μL index 1 primer, 5 μL index 2 primer, 10 μL molecular grade PCR water, and 5 μL amplified genomic DNA. PCR conditions were: denaturation for 3 min at 95 °C, ligation for 8 cycles of 30 s at 95 °C, 30 s at 55 °C, 30 s at 72 °C, and a final extension for 5 min at 72 °C. These products were checked for integrity on a 1% agarose gel and then quantified using the Qubit dsDNA HS Assay Kit (Thermofisher, Waltham, MA, United States). Equal concentrations of each sample were pooled and the library pool was sequenced on two Illumina MiSeq runs using 2 × 250 paired end technology at the Genomics and Microbiology Research Lab of the North Carolina Museum of Natural Sciences.

### Sequence processing

Sequence reads were quality filtered and processed using the program Quantitative Insights Into Microbial Ecology 2 (vQIIME2-2020.2) [75, 76]. Demultiplexed forward reads from two Illumina MiSeq 2 × 250 platform runs were imported and filtered using the following criteria: minimum PHRED score of 4, a maximum of three consecutive low-quality PHRED scores observed before truncation, and zero ambiguous base calls (N’s) within the sequence. Only forward reads were used for analysis due to the poor quality of reverse reads for both Illumina runs [16, 77, 78]. Quality filtered sequences were trimmed to 220 bp and clustered into sub-operational taxonomic units (sOTUs) using the Deblur workflow [16, 79], hereafter referred to solely as OTUs. Taxonomy was then assigned by aligning sequences with the Greengenes 13_8 99% database (Naïve Bayes classifier trained on the 515f/806r region) and a phylogenetic tree was built using the fasttree algorithm [80]. Sequencing depth per sample ranged from 5213 to 28,676, equating to a fivefold increase. Due to this relatively low difference in library sizes we did not rarefy as that would not improve our false discovery rate and might introduce biases [81, 82]. The final OTU table was filtered to keep OTUs that had at least two representative sequences and that were detected in at least 2% of samples using the phyloseq package in R (v4.0.3) [83] (phyloseq 1.32.0) [84]. All subsequent analyses were conducted in R. Sequences were uploaded to NCBI’s SRA database and can be accessed via BioProject ID PRJNA705959.

### Data analysis

Descriptive summaries of microbial taxa for all samples were obtained using the R package ‘phyloseq’ [84]. Differences in species richness (SR) and Faith’s phylogenetic diversity (PD) were determined by ANOVAs with a significance threshold of 0.05. Beta diversity across adults, nests, tadpoles in the nest, leaves, water inside the terrarium, water from the pond, and tadpoles after pond water interaction was tested with permutational multivariate analysis of variance (PERMANOVA) using the non-parametric *adonis* function from the R package ‘vegan’ with 999 random permutations [85]. A triangulation test was run in MEGAN v6.19.2 [86] to determine distributions between replicates. All replicates for adults and tadpoles were first analyzed via PERMANOVA along with the “strata” argument where appropriate (e.g., when testing differences for sex, body location was accounted for using strata) to determine any species level effects. Afterwards, data for the three species (n = 3 nests; one nest per species for *Polypedates leucomystax*, *P. macrotis*, and *P. otilophus*) were combined and each replicate set were analyzed separately for differences between categories (see Additional file 1: Table S1).

SourceTracker (v0.9.1) analysis was applied to tadpoles in the nest and tadpoles after pond water interaction to discern microbial sources on the tadpole skin microbiome [87]. Then, vertical versus environmental transmission of microbes to tadpoles before and after pond water interaction was examined first by binning shared OTUs using the R package ‘ggVennDiagram’ (v1.2.1) [88]. We then used differential abundance analyses to identify OTUs with different abundances between categories (e.g., tadpole microbiomes, both from inside the foam nest and from the pond were compared to the microbiome of adults, the nest inside, the nest outside, and the pond water). The OTU count matrix was used to test differences between categories running six statistical models with R package DESeq2 [89]. Count data was normalized and fitted to GMLs using estimateSizeFactors() with the option postcounts (OTUs with 0 counts in same of samples), estimateDispersions(), nbinomWaldTest(), and results() defining each pairwise contrasts (tadpole-nest vs. adult, tadpole-nest vs. nest-inside, tadpole-nest vs. nest-outside, tadpole-pond vs. tadpole-nest, tadpole-pond vs. water, and tadpole-pond vs. adult). We sorted the results of the contrasts using adjust *p* values and change in abundance (Fold change). Significant differential abundance OTUs were defined as the OTUs with 1 or more units of fold change and an adjust *p* value < 0.05. Those OTUs were annotated using our taxonomy table from the biom file.

## Supplementary Information

**Additional file 1.** **Table S1.** Pairwise PERMANOVA results for each set of variables for Jaccard (presence/absence), Bray-Curtis (abundance), unweighted UniFrac (phylogenetic relationships), and weighted UniFrac (phylogenetic relationships and abundance) distance matrices. **Figure S1.** Taxonomic bar plots depicting the bacterial community composition (by genera) of amphibian adults and tadpoles (by species).  **Table S2.** List of taxonomic designations for novel amphibian associated genera recovered from the skin of the Bruneian frogs used in this study and not known from a global dataset of amphibian specific bacterial microbiome data. **Table S3.** List of differentially expressed OTU’s associated with the cutaneous tadpole microbiome that had a magnitude of change greater than 10 units. **Figure S2.** Volcano plots comparing differential abundance of OTUs during vertical and environmental transmission. **Table S4.** Voucher specimen accession numbers and associated field ID numbers.

## Data Availability

Demultiplexed DNA sequences are available at the NCBI’s SRA: PRJNA705959. The datasets generated and/or analyzed during the current study are available in the smcgblaser/Amphib_microbiome_Brunei: Bornean Frog Microbiome Repo Release 1 repository, http://doi.org/10.5281/zenodo.4663442.
