# Supplementary material for: Early life skin microbial trajectory as a function of vertical and environmental transmission in Bornean foam-nesting frogs
Source: Anim Microbiome. 2021 Dec 20;3:83. doi: 10.1186/s42523-021-00147-8 (PMC8686334; doi:10.1186/s42523-021-00147-8)
Supplement: Supplementary file 1 — Additional file 1. Table S1. Pairwise PERMANOVA results for each set of variables for Jaccard (presence/absence), Bray-Curtis (abundance), unweighted UniFrac (phylogenetic relationships), and weighted UniFrac (phylogenetic relationships and abundance) distance matrices. Figure S1. Taxonomic bar plots depicting the bacterial community composition (by genera) of amphibian adults and tadpoles (by species). Table S2. List of taxonomic designations for novel amphibian associated genera recovered from the skin of the Bruneian frogs used in this study and not known from a global dataset of amphibian specific bacterial microbiome data. Table S3. List of differentially expressed OTU’s associated with the cutaneous tadpole microbiome that had a magnitude of change greater than 10 units. Figure S2. Volcano plots comparing differential abundance of OTUs during vertical and environmental transmission. Table S4. Voucher specimen accession numbers and associated field ID numbers. [file 42523_2021_147_MOESM1_ESM.docx]

**Supplemental Materials**

Table 1. Pairwise PERMANOVA results for each set of variables for Jaccard (presence/absence), Bray-Curtis (abundance), unweighted UniFrac (phylogenetic relationships), and weighted UniFrac (phylogenetic relationships and abundance) distance matrices. Each REP includes all biological replicates and one of the replicates for nest-in and nest-out. For adults REP1 = cloaca, REP2 = dorsum, REP3 = venter. X’s represent significant differences between variables (p<0.05) and dots depict rows where only one set of replicates for those variables were significantly different. Differences between variables were only considered significant overall if two replicates came back as different. Any results that could be rounded up to have a p-value of 0.05 or higher were not considered significant.

|  | **Jaccard** | | | **Bray-curtis** | | | **Unweighted UniFrac** | | | **Weighted UniFrac** | | |
| --- | --- | --- | --- | --- | --- | --- | --- | --- | --- | --- | --- | --- |
|  | **REP1** | **REP2** | **REP3** | **REP1** | **REP2** | **REP3** | **REP1** | **REP2** | **REP3** | **REP1** | **REP2** | **REP3** |
| **Adult--leaf** |  |  |  |  |  |  |  |  |  |  |  |  |
| **Adult--nest-in** | • |  |  | • |  |  | x | x | x | • |  |  |
| **Adult--nest-out** |  |  |  |  |  |  |  |  |  |  |  |  |
| **Adult--tadpole-nest** |  |  |  |  | x | x |  |  |  |  |  |  |
| **Adult--tadpole-pond** | x | x | x | x | x | x | x | x | x | x | x | x |
| **Adult--water-pond** | x | x | x | x | x | x | x | x | x | x | x | x |
| **Adult--water-terrarium** | • |  |  | • |  |  | • |  |  | x | x |  |
| **Leaf--nest-in** |  |  |  |  |  |  |  |  |  |  |  |  |
| **Leaf--nest-out** |  |  |  |  |  |  |  |  |  |  |  |  |
| **Leaf--tadpole-nest** | x | x | x | x | x | x | x | x | x | x | x | x |
| **Leaf--tadpole-pond** | x | x | x | x | x | x | x | x | x | x | x | x |
| **Leaf--water-pond** | x | x | x | x | x | x | x | x | x | x | x | x |
| **Leaf--water-terrarium** |  |  |  | x | x | x |  |  |  | x | x | x |
| **Nest-in--Nest-out** |  |  |  |  | • |  |  |  |  |  |  |  |
| **Nest-in--tadpole-nest** | x | x | x | x | x | x | x | x | x | x | x | x |
| **Nest-in--tadpole-pond** | x | x | x | x | x | x | x | x | x |  |  |  |
| **Nest-in--water-pond** | x | x | x | x | x | x | x | x | x | x | x | x |
| **Nest-in--water-terrarium** |  |  | • |  |  |  |  |  |  |  |  |  |
| **Nest-out--tadpole-nest** |  |  |  |  |  |  |  |  |  |  |  |  |
| **Nest-out--tadpole-pond** | x | x |  | x | x | x | x | x | x |  |  |  |
| **Nest-out--water-pond** | x | x | x | x | x | x | x | x |  | x | x |  |
| **Nest-out--water-terrarium** |  |  |  |  |  |  |  |  |  | x | x | x |
| **Tadpole-nest--tadpole-pond** | x | x | x | x | x | x | x | x | x | x | x | x |
| **Tadpole-nest--water-pond** | x | x | x | x | x | x | x | x | x | x | x | x |
| **Tadpole-nest--water-terrarium** | x | x | x | x | x | x | x | x | x | x | x | x |
| **Tadpole-pond--water-pond** | x | x | x | x | x | x | x | x | x | x | x | x |
| **Tadpole-pond--water-terrarium** | x | x | x | x | x | x | x | x | x | x | x | x |
| **Water-enclosure--water-terrarium** | x | x | x | x | x | x | x | x | x | x | x | x |


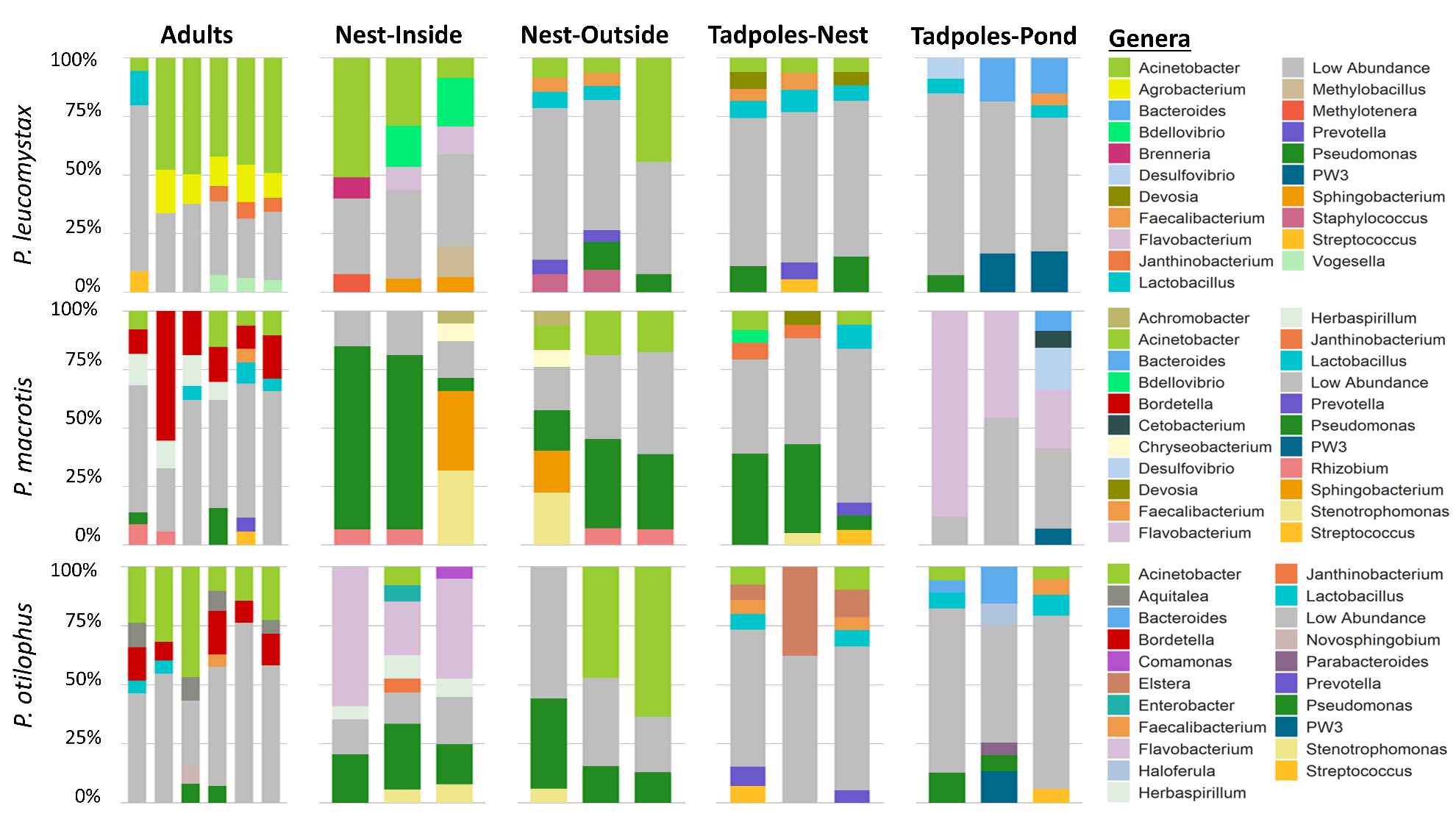


Figure 1. Taxonomic bar plots depicting the bacterial community composition (by genera) of amphibian adults and tadpoles (by species). Adult replicates are arranged by sex (females are the left three bars and males are the right three) and body location (cloaca, dorsum, venter). Genera with <5% abundance were grouped into a ‘Low Abundance’ category indicated by the gray color in the figure. Low abundance genera constitute a large portion of the microbial community.

Table 2. List of taxonomic designations for novel amphibian associated genera recovered from the skin of the Bruneian frogs used in this study and not known from a global dataset of amphibian specific bacterial microbiome data [21].

| **Kingdom** | **Phylum** | **Class** | **Order** | **Family** | **Genus** | **Species** |
| --- | --- | --- | --- | --- | --- | --- |
| Bacteria | Actinobacteria | Actinobacteria | Actinomycetales | Nocardioidaceae | Marmoricola | NA |
| Bacteria | Actinobacteria | Actinobacteria | Actinomycetales | Nocardioidaceae | Marmoricola | bigeumensis |
| Bacteria | Actinobacteria | Actinobacteria | Actinomycetales | Nocardioidaceae | Marmoricola | NA |
| Bacteria | Actinobacteria | Actinobacteria | Actinomycetales | Nocardioidaceae | Marmoricola | bigeumensis |
| Bacteria | Actinobacteria | Actinobacteria | Actinomycetales | Nocardioidaceae | Marmoricola | aequoreus |
| Bacteria | Bacteroidetes | Bacteroidia | Bacteroidales | Porphyromonadaceae | Macellibacteroides | fermentans |
| Bacteria | Bacteroidetes | Cytophagia | Cytophagales | Cyclobacteriaceae | Fontibacter | flavus |
| Bacteria | Firmicutes | Bacilli | Bacillales | Planococcaceae | Chryseomicrobium | imtechense |
| Bacteria | Firmicutes | Clostridia | Clostridiales | Clostridiaceae | 02d06 | NA |
| Bacteria | Firmicutes | Clostridia | Clostridiales | Clostridiaceae | 02d06 | NA |
| Bacteria | Firmicutes | Clostridia | Clostridiales | Ruminococcaceae | Gemmiger | formicilis |
| Bacteria | Firmicutes | Clostridia | Clostridiales | Ruminococcaceae | Gemmiger | formicilis |
| Bacteria | Firmicutes | Clostridia | Clostridiales | Ruminococcaceae | Gemmiger | formicilis |
| Bacteria | Firmicutes | Clostridia | Clostridiales | Lachnospiraceae | Pseudobutyrivibrio | NA |
| Bacteria | Proteobacteria | Alphaproteobacteria | Rickettsiales | mitochondria | Carica | papaya |
| Bacteria | Proteobacteria | Betaproteobacteria | Burkholderiales | Oxalobacteraceae | Duganella | nigrescens |
| Bacteria | Proteobacteria | Betaproteobacteria | Burkholderiales | Oxalobacteraceae | Duganella | nigrescens |
| Bacteria | Proteobacteria | Betaproteobacteria | Burkholderiales | Alcaligenaceae | Pusillimonas | noertemannii |
| Bacteria | Proteobacteria | Betaproteobacteria | Burkholderiales | Alcaligenaceae | Bordetella | petrii |
| Bacteria | Proteobacteria | Betaproteobacteria | Burkholderiales | Alcaligenaceae | Bordetella | petrii |
| Bacteria | Proteobacteria | Betaproteobacteria | Burkholderiales | Comamonadaceae | Sphaerotilus | natans |
| Bacteria | Proteobacteria | Betaproteobacteria | Burkholderiales | Comamonadaceae | Inhella | inkyongensis |
| Bacteria | Proteobacteria | Gammaproteobacteria | Xanthomonadales | Sinobacteraceae | Solimonas | soli |
| Bacteria | Proteobacteria | Alphaproteobacteria | Rhodospirillales | Rhodospirillaceae | Reyranella | massiliensis |
| Bacteria | Proteobacteria | Alphaproteobacteria | Rhodospirillales | Rhodospirillaceae | Reyranella | massiliensis |
| Bacteria | Proteobacteria | Alphaproteobacteria | Rhodospirillales | Rhodospirillaceae | Reyranella | massiliensis |
| Bacteria | Proteobacteria | Alphaproteobacteria | Rhodospirillales | Rhodospirillaceae | Reyranella | massiliensis |
| Bacteria | Proteobacteria | Alphaproteobacteria | Rhodospirillales | Rhodospirillaceae | Reyranella | massiliensis |
| Bacteria | Proteobacteria | Alphaproteobacteria | Rhizobiales | Aurantimonadaceae | Aurantimonas | NA |
| Bacteria | Proteobacteria | Alphaproteobacteria | Rhizobiales | Rhizobiaceae | Ensifer | adhaerens |
| Bacteria | Proteobacteria | Alphaproteobacteria | Rhizobiales | Hyphomicrobiaceae | Rhodomicrobium | vannielii |

Table 3. List of differentially expressed OTUs associated with the cutaneous tadpole microbiome that had a magnitude of change greater than 10 units.

| **Kingdom** | **Phylum** | **Class** | **Order** | **Family** | **Genus** | **Species** | **Base mean abundance** | **Log2**  **fold change** | **Adjust p-value** |
| --- | --- | --- | --- | --- | --- | --- | --- | --- | --- |
| Bacteria | Proteobacteria | Alphaproteobacteria | Rhodospirillales | Rhodospirillaceae | Elstera | litoralis | 49.73 | 34.71 | 3.09E-07 |
| Bacteria | Proteobacteria | Betaproteobacteria | Burkholderiales | Comamonadaceae | Hylemonella | NA | 18.77 | 32.90 | 2.79E-20 |
| Bacteria | Proteobacteria | Alphaproteobacteria | Rhizobiales | NA | NA | NA | 5.58 | 29.68 | 6.14E-07 |
| Bacteria | Proteobacteria | Deltaproteobacteria | Bdellovibrionales | Bdellovibrionaceae | Bdellovibrio | bacteriovorus | 7.65 | 28.75 | 2.66E-05 |
| Bacteria | Proteobacteria | Betaproteobacteria | Burkholderiales | Oxalobacteraceae | NA | NA | 24.52 | 28.46 | 3.60E-10 |
| Bacteria | Bacteroidetes | Saprospirae | Saprospirales | Chitinophagaceae | NA | NA | 3.48 | 25.94 | 1.81E-04 |
| Bacteria | Proteobacteria | Alphaproteobacteria | Rhizobiales | NA | NA | NA | 5.67 | 23.76 | 0.00069 |
| Bacteria | Proteobacteria | Alphaproteobacteria | Rhizobiales | NA | NA | NA | 3.44 | 22.02 | 0.00178 |
| Bacteria | Verrucomicrobia | Verrucomicrobiae | Verrucomicrobiales | Verrucomicrobiaceae | NA | NA | 3.11 | 21.41 | 0.00246 |
| Bacteria | Proteobacteria | Betaproteobacteria | Burkholderiales | Comamonadaceae | NA | NA | 4.54 | 21.13 | 0.00282 |
| Bacteria | Proteobacteria | Alphaproteobacteria | Rhizobiales | Rhizobiaceae | Agrobacterium | NA | 4.90 | 19.27 | 1.50E-14 |

Fig. 2. Volcano plots comparing differential abundance of OTUs during vertical and environmental transmission. A-C) Skin microbiome of the tadpole in the nest is compared to the adult, the nest interior, and the nest exterior. Green color represents OTUs that are highly abundant (positive values) in the skin microbiome of the tadpoles. D) Results of the comparison between tadpoles inside the nest and tadpoles in the pond. E-F) Results from the comparisons between tadpoles in the pond and pond water, and adult frog microbiome, respectively.

Table 4. Voucher specimen accession numbers and associated field ID numbers. All specimens are accessioned and curated at the North Carolina Museum of Natural Sciences in Raleigh, North Carolina.

| Species | Life Stage | NCSM No. | Field Collection ID |
| --- | --- | --- | --- |
| *Polypedates leucomystax* | Adult | 104918 | DSM2747 |
|  | Adult | 104919 | DSM2748 |
|  | Tadpoles | 104948 | DSM2766 |
| *Polypedates macrotis* | Adult | 104922 | DSM2622 |
|  | Adult | 104923 | DSM2623 |
|  | Tadpoles | 104945 | DSM2763 |
| *Polypedates otilophus* | Adult | 104927 | DSM2659 |
|  | Adult | 104928 | DSM2660 |
|  | Tadpoles | 104947 | DSM2765 |
